# Investigation of the Compatibility and Damping Performance of Graphene Oxide Grafted Antioxidant/Nitrile-Butadiene Rubber Composite: Insights from Experiment and Molecular Simulation

**DOI:** 10.3390/polym14040736

**Published:** 2022-02-14

**Authors:** Meng Song, Xiulin Yue, Chaokang Chang, Fengyi Cao, Guomin Yu, Xiujuan Wang

**Affiliations:** 1 School of Materials and Chemical Engineering, Zhongyuan University of Technology, Zhengzhou 450007, China; yuelll333@126.com (X.Y.); changchaokang@126.com (C.C.); caofengyi0513@126.com (F.C.); yuguomin1129@126.com (G.Y.); 2 Key Laboratory of Rubber-Plastics, Ministry of Education/Shandong Provincial Key Laboratory of Rubber-Plastics, Qingdao University of Science & Technology, Qingdao 266042, China

**Keywords:** molecular dynamics simulation, damping performance, nitrile-butadiene rubber, graphene oxide, antioxidant 4010NA

## Abstract

Rubber damping materials are widely used in electronics, electrical and other fields because of their unique viscoelasticity. How to prepare high-damping materials and prevent small molecule migration has attracted much attention. Antioxidant 4010NA was successfully grafted onto graphene oxide (GO) to prepare an anti-migration antioxidant (GO-4010NA). A combined molecular dynamics (MD) simulation and experimental study is presented to investigate the effects of small molecules 4010NA, GO, and GO-4010NA on the compatibility and damping properties of nitrile-butadiene rubber (NBR) composites. Differential scanning calorimetry (DSC) results showed that both 4010NA and GO-4010NA had good compatibility with the NBR matrix, and the *T_g_* of GO-4010NA/NBR composite was improved. Dynamic mechanical analysis (DMA) data showed that the addition of GO-4010NA increased the damping performance of NBR than that of the addition of 4010NA. Molecular dynamics (MD) simulation results show GO-4010NA/NBR composites have the smallest free volume fraction (FFV) and the largest binding energy. GO-4010NA has a strong interaction with NBR due to the forming of hydrogen bonds (H-bonds). Grafting 4010NA onto GO not only inhibits the migration of 4010NA but also improves the damping property of NBR matrixes. This study provides new insights into GO grafted small molecules and the design of high-damping composites.

## 1. Introduction

Rubber damping materials are widely used in electronics, electrical, aerospace, and automobiles for vibration and noise reduction due to their unique viscoelastic properties [1,2,3]. As some special fields have higher requirements for rubber damping materials, the preparation of high-performance damping materials has become a research hotspot. In recent years, the preparation, and research of organic hybrid damping materials have attracted much attention in the field of rubber damping [4,5]. Rubber damping materials with a dynamic hydrogen bond (H-bond) network can be prepared by adding small molecular compounds with polar functional groups, such as hydroxyl and amino into the polar rubber matrix, which can significantly improve the damping properties of the material [6,7]. In the previous studies, researchers often used nitrile-butadiene rubber (NBR) as the polar rubber matrix and added hindered phenol antioxidants (such as AO-80 or AO-60, etc.) to prepare organic hybrid damping materials with high-damping performance [8,9,10].

NBR has good damping performance due to the strong polarity of the -CN functional groups. However, NBR molecular chain contains a large number of unsaturated carbon-carbon double bonds, which is easy to age in heat, oxygen, ozone, light, and other external conditions, thus shortening the service life and affecting the damping effect. At present, adding amine antioxidants into a rubber matrix is one of the easiest ways to prevent rubber aging. For most rubbers, amine antioxidants are more effective in preventing long-term oxidative degradation [11]. Many researchers improve the phenomenon of rubber aging easily by adding a small molecular antioxidant, but the antioxidant tends to migrate from the rubber matrix due to their low molecular weight, thus affecting the damping effect, resulting in shortened service life and environmental pollution [12,13,14]. At present, there are two solutions: the first is to improve the molecular weight of antioxidants [15,16]; the second is to graft antioxidants to the polymer chain or filler surface [17,18,19,20,21], which is an effective method for anti-migration of antioxidants.

Graphene oxide (GO) is a two-dimensional carbon material, arranged in a honeycomb shape, with an extremely high specific surface area and high stability. Compared with graphene, its surface contains a large number of oxygen-containing functional groups, such as hydroxyl (-OH), carboxyl (-COOH), and epoxy functional groups (-CH (O) CH-), which make GO have excellent mechanical, thermal, and electrical properties [22,23,24,25] and more practical application value and broad prospects. It can usually show a significant enhancement effect on some polar rubbers [26,27,28,29].

Grafting antioxidants on the surface of GO is an effective method to reduce the migration of antioxidants. At present, experimental studies have been carried out to graft antioxidants onto GO and other fillers. For example, Zhong et al. [30] modified GO with the antioxidant p-phenylenediamine (PPD), and added the modified GO into the NBR matrix, which significantly improved the thermal stability of the rubber matrix. Yao et al. [31] functionalized GO nanosheets through an acyl chloride reaction and then reacted the product with hindered phenol (HP) to obtain GO-g-HP with excellent anti-aging effects. Zhong et al. [32] grafted antioxidant 4020 to the surface of GO, which showed better anti-migration performance in styrene-butadiene rubber (SBR) than free antioxidants. By grafting the antioxidant onto the GO surface, the antioxidant can be fixed to prevent the migration in the rubber matrix, and the dispersity in the rubber can be improved. Generally, amine antioxidants contain polar functional groups, the addition of antioxidants can also form an H-bonds network with a polar rubber matrix, which can improve the damping and aging properties of rubber.

At present, there are few studies on the microstructure of GO grafted antioxidants from the molecular level, and the microscopic mechanism of adding GO grafted antioxidants into rubber to prevent migration and improve the damping performance of the rubber matrix. Molecular dynamics (MD) simulation, as a novel, practical, and powerful theoretical tool, can not only explain phenomena and processes that are difficult to be considered in traditional experiments but also predict experimental results. MD simulation has been widely used to study the relationship between microstructure and properties of materials [8,10,33].

In this study, antioxidant N-isopropyl-N’-phenyl-p-phenylenediamine (4010NA) was selected as a damping additive. NBR was selected as a polar rubber matrix. Small molecule 4010NA contains imino, which is easy to form H-bonds with NBR. Firstly, antioxidant 4010NA was grafted onto GO to prepare an anti-migration antioxidant (GO-4010NA). Then, the microstructure, compatibility, and damping properties of GO-4010NA/NBR composites were investigated by combining the experiment and MD simulation. For comparative analysis, control systems including NBR, GO/NBR, 4010NA/NBR, GO/4010NA/NBR were also studied. We expect to establish correlations between the microstructures and the damping properties.

## 2. Experimental Section

### 2.1. Materials

NBR with an acrylonitrile mass fraction of 41% (N220S) was purchased from Japan synthetic rubber co., Ltd. (Tokyo, Japan). GO was provided by Sixth Element Materials Technology Co., Ltd. (Changzhou, China). Antioxidant N-isopropyl-N′-phenyl-p-phenylenediamine (4010NA) was obtained from Shangshun Chemical (Heze, China). N, N-dimethylformamide (anhydrous grade, 99.8%), and sulfoxide chloride were purchased from Aladdin (Shanghai, China). All other raw materials are commercially available industrial products.

### 2.2. Preparation of NBR Composites

#### 2.2.1. Synthesis of Anti-Migration Antioxidant GO-4010NA

**Figure 1:**
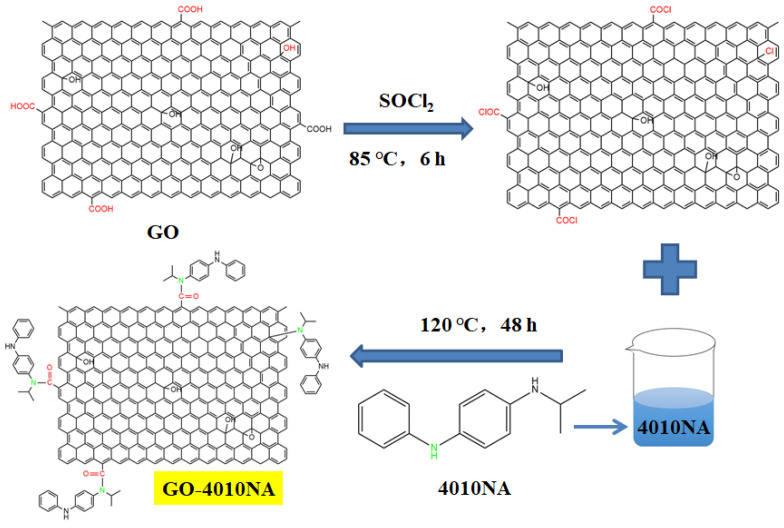
The synthesis route of damping additives.

The steps for the synthesis of anti-migration antioxidants are shown in Figure 1. First, 1 g GO was weighed and dissolved in 150 mL sulfoxide chloride (SOCl_2_). The reaction was performed at room temperature for 1 h under ultrasound, and then at 85 °C for 6 h, after which SOCl_2_ was removed by rotary steaming. Then it was added to 100 mL DMF for an ultrasound for 1 h, 5 g 4010NA was dissolved in 200 mL DMF and added to GO solution for reflux reaction at 120 °C for 48 h. Then the reaction solution was pumped and filtered, the solid product was poured into anhydrous ethanol for soaking for 12 h. During the extraction and filtration process, anhydrous ethanol was used for washing 4–6 times. Finally, the solid product was dried in a 40 °C vacuum oven for 12 h to obtain GO-4010NA.

#### 2.2.2. Preparation of NBR Composites

The experimental formulae are shown in Table 1. First, 100 g NBR was plasticized at room temperature for 3 min on a two-roll mill. Then 4010NA (0.1 g), GO (1 g), GO (1 g) and 4010NA (0.1 g), GO-4010NA (1 g) were added to the above NBR, respectively. Then, the pure NBR and the four groups of samples were mixed at room temperature for 5 min. Rubber additives are added to the above samples, including 5 phr of zinc oxide (ZnO), 1 phr of stearic acid (SA), 0.5 phr of promoter D (diphenyl guanidine), 0.5 phr of promoter DM (dibenzothiazole disulfide), 0.2 phr of promoter TMTD (tetramethyl thiuram disulfide), 2 phr of sulfur hit the triangle package and cutting material, mixing evenly, mixing for 10 min. Finally, the samples were hot-pressed and vulcanized for 15 min at 15 MPa and 150 °C, and then cooled naturally to room temperature.

### 2.3. Characterization

Fourier transform infrared spectra (FTIR) were obtained from a Nicolet iS50 spectrometer made by Thermo Scientific Inc. (Waltham, MA, USA) within the 4000–400 cm^−1^. The potassium bromide pellet technique and attenuated total reflection (ATR) technique were applied to powder antioxidants and NBR composites, respectively.

X-ray photoelectron spectroscopy (XPS) spectra were determined by an ESCALAB 250 Xi made by Thermo Fischer Inc. (Waltham, MA, USA). Excitation source was Al-Ka ray (HV = 1486.6 eV), operating voltage was 12.5 kV.

Differential scanning calorimetry (DSC) measurements were performed using a Netzsch DSC 200F3 calorimeter made by NETZSCH Scientific Instruments Trading Ltd. (Germany) under a nitrogen atmosphere. Samples were heated at a rate of 20 °C/min from room temperature to 100 °C, kept at 100 °C for 5 min, then cooled to −80 °C at a rate of 20 °C/min, and then heated to 180 °C at a rate of 10 °C/min.

Dynamic mechanical analysis (DMA) measurements were obtained by a Q800 dynamic mechanical analyzer made by TA instruments Inc. (New Castle, DE, USA). The samples’ length, width, and thickness were 20 mm, 10 mm, and approximately 2 mm, respectively. The temperature dependence of the loss factor (tan *δ*) was measured from −50 °C to 150 °C at a constant frequency of 10 Hz and a heating rate of 3 °C/min in tension mode.

## 3. Model and Simulation Details

Materials Studio (MS) 7.0 software was used to reveal the effects of different fillers on NBR composites from the microscopic aspect, providing theoretical guidance for the experimental study of GO graft antioxidants.

The MD simulation was carried out using the Forcite and Amorphous Cell modules. During the simulation, the Andersen thermostat is used for temperature control, and the Berendsen barostat is used for pressure control. In the MD model, COMPASS force field is adopted. In COMPASS force field, the total energy *E_T_* of the system is the sum of bond energy and non-bond energy, and the calculation formula is as follows [8].

(1) $$E_{T}=E_{b}+E_{\theta }+E_{\phi }+E_{\chi }+E_{cross}+E_{ele}+E_{vdW}$$

In the formula, *E_b_* is the bond stretching energy, *E_θ_* is the bond Angle bending energy, *E_φ_* is the dihedral Angle torsion energy, *E_χ_* is the out-of-plane energy, *E_cross_* is the cross term interaction energy, *E_ele_* is electrostatic interaction energy and *E_vdW_* is van der Waals interaction energy. The sum of the first five energies is the bond energy, and the sum of the last two energies is the non-bond energy.

### 3.1. Construction of GO Model

**Figure 2:**
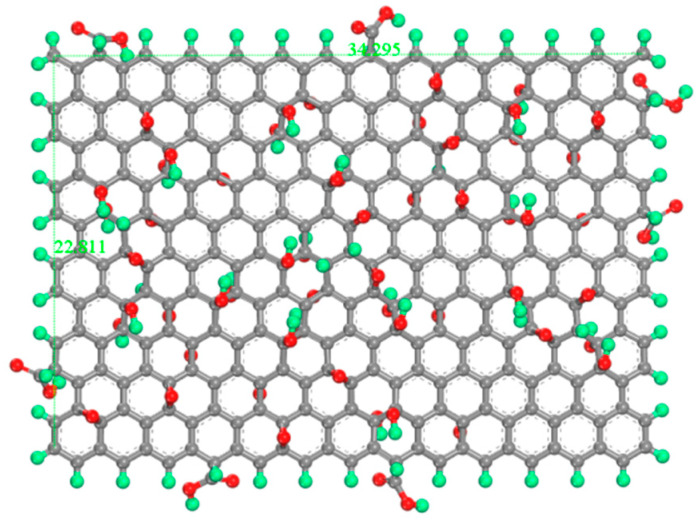
Molecular models for GO. The green atom is H, the gray atom is C, and the red atom is O.

The GO model adopts the classic Lerf–Klinowski model: C_10_O_1_(OH)_1_(COOH)_0.5_ [34,35,36], which represents the results of the standard oxidation process. GO model is C_297_O_28_(OH)_28_(COOH)_14_, hydroxyl and epoxy groups are randomly distributed on the surface of GO, carboxyl groups are distributed on the edge of GO, and the oxidation degree is 21.27%. The length and width of the GO model are 34.295 Å and 22.811 Å, as shown in Figure 2.

### 3.2. Construction of Composite System Model

**Figure 3:**
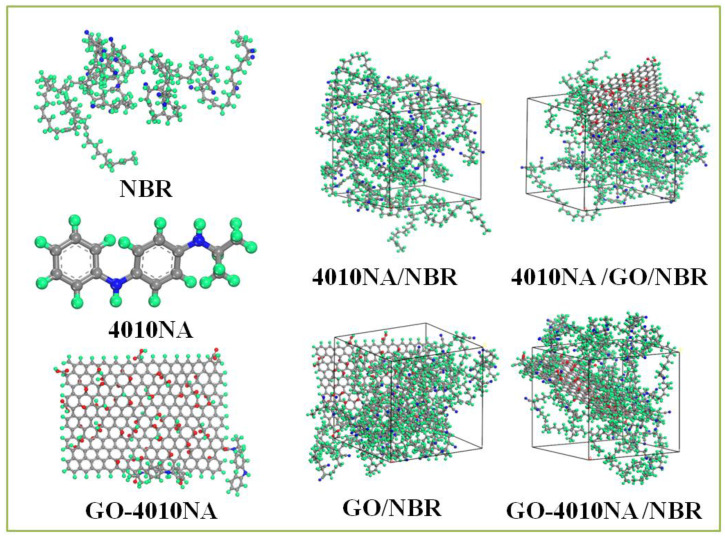
The construction of the 4010NA/NBR, GO/NBR, 4010NA/GO/NBR, and GO-4010NA/NBR composites amorphous cells (The green atom is H, the gray atom is C, the blue atom is N, and the red atom is O).

NBR is a polar rubber, because the molecular chain contains polar group nitrile (-NH) and the double bond structure has good performance and is widely used. Therefore, we selected NBR and constructed pure NBR (NBR molecular chain consists of 50 repeating units, 41% acrylonitrile content, and 59% butadiene content). 4010NA/NBR, GO/NBR, 4010NA/GO/NBR and GO-4010NA/NBR composite models were also constructed. The 4010NA/NBR composite has 4 NBR polymer chains and 2 4010NA small molecules, the GO/NBR composite has 4 NBR polymer chains and 1 GO, and the 4010NA/GO/NBR composite has 4 NBR polymer chains, 2 4010NA small molecules and 1 GO. The GO-4010NA/NBR composite has 4 NBR polymer chains and one GO-4010NA, as shown in Figure 3.

After the periodic cells are constructed, a series of simulations are needed in order to keep the system in equilibrium. First, the energy of amorphous cells is optimized by 2 million steps by geometric optimization with a convergence value of 1.0 × 10^−5^ kcal/mol/Å. The optimized cells are annealed from 200 K to 500 K with 200 annealing cycles. Then, the dynamics simulation was performed by an NVT ensemble (constant atomic number, constant volume, and constant temperature) at room temperature 298 K and the time length was set at 1000 ps. Finally, the dynamics simulation of the NPT ensemble (constant atomic number, constant pressure, and constant temperature) was carried out. The temperature is set at room temperature 298 K, the pressure is set at 0.1 MPa, and the time length is set at 1000 ps. The relevant physical parameters are calculated for the system reaching perfect equilibrium.

## 4. Results and Discussion

### 4.1. Structure Analysis of the Synthetic Antioxidant GO-4010NA

**Figure 4:**
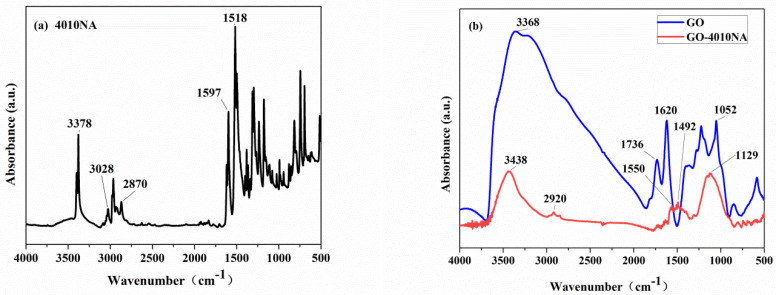
FTIR spectrum of (**a**) 4010NA and (**b**) GO and GO-4010NA.

The structure of the anti-migration damping agent (GO-4010NA) was characterized by FTIR, as shown in Figure 4. From Figure 4a, the peak at a wavenumber of 3378 cm^−1^ is attributed to -NH- vibration. The peaks at wavenumber 1597 cm^−1^ and 1518 cm^−1^ are attributed to the benzene ring vibration [36]. In Figure 4b, pure GO has a broad absorption peak in the wavenumber range 2500–3750 cm^−1^, which belongs to the -OH vibrations. The carbonyl group -C=O has a strong infrared absorption peak at 1736 cm^−1^ [10]. Compared to GO, the carbonyl peak is absent, and the hydroxyl peak is weakened in the GO-4010NA spectra. In addition, new peaks appear at 1550 cm^−1^ and 1492 cm^−1^, which belong to the benzene ring vibration, indicating that 4010NA was grafted to GO.

**Figure 5:**
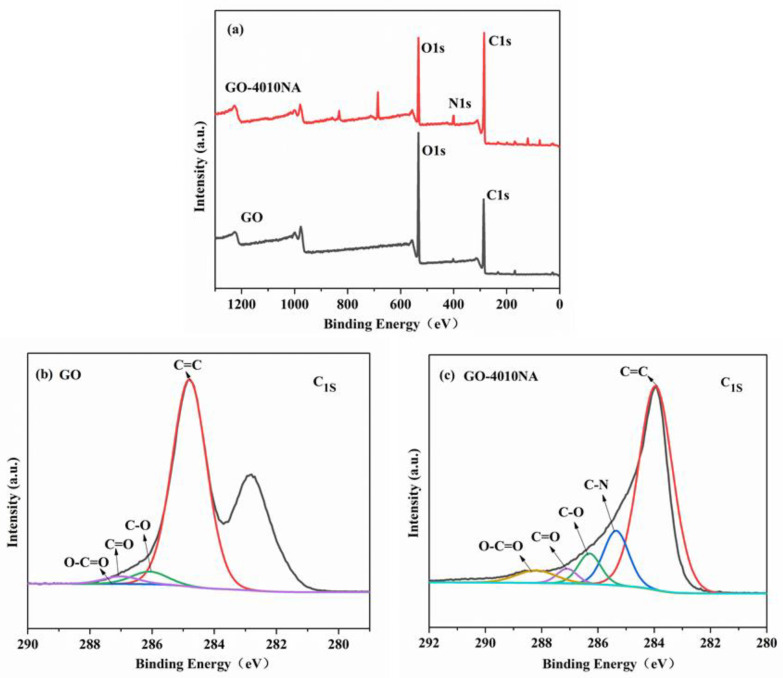
(**a**) XPS survey scans for GO and GO-4010NA, XPS C 1s core-level spectra for (**b**) GO and (**c**) GO-4010NA.

To further illustrate whether 4010NA was grafted to GO, an XPS test was performed as shown in Figure 5. From Figure 5b, for GO, two peaks are detected at 284.8 eV (C1s) and 534.6 eV (O1s). For GO-4010NA from Figure 5c, in addition to strong signals of C1s and O1s, N1s signals were also found at 398.2 eV, indicating that 4010NA molecules were linked to the GO layer. All the above results indicate that the small molecule 4010NA was successfully grafted onto GO.

### 4.2. FTIR Analysis of NBR Composites

**Figure 6:**
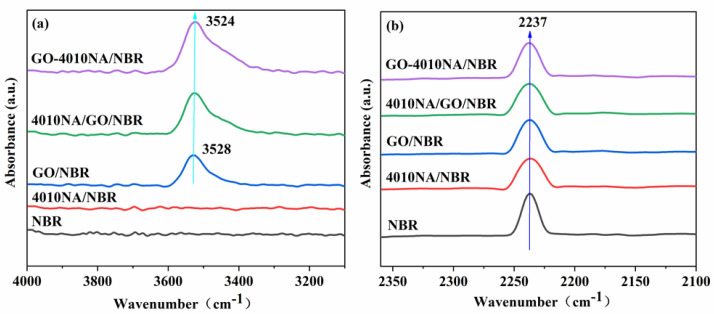
FTIR spectrum of (**a**) NBR composites with different fillers added illustrating the hydroxyl -OH stretching vibration region and (**b**) NBR composites with different fillers added illustrating the the nitrile -CN stretching vibration region.

Figure 6 shows the FTIR spectrum of NBR composites with different contents. The telescopic vibration of -OH is generally at the wavenumber range 3125–3704 cm^−1^. In Figure 6a, the spectrum of the neat NBR and 4010NA/NBR hardly reveals any absorbance band in the wavenumber range 3200–4000 cm^−1^ [8], whereas the other three systems containing GO showed significant peaks at 3400–3600 cm^−1^. Compared with GO/NBR and 4010NA/GO/NBR composites, the -OH peak in GO-4010NA/NBR composites becomes stronger and shows a red shift, which is mainly attributed to the formation of hydrogen bonds in GO-4010NA/NBR composites. It can be seen from Figure 6b that the stretching vibration peak in the range of 2220–2260 cm^−1^ is -CN groups. After the addition of GO-4010NA, the -CN peak value is significantly weakened compared with pure NBR, indicating that many -CN groups in the composite are involved in the formation of hydrogen bonds.

### 4.3. DSC Analysis NBR Composites

**Figure 7:**
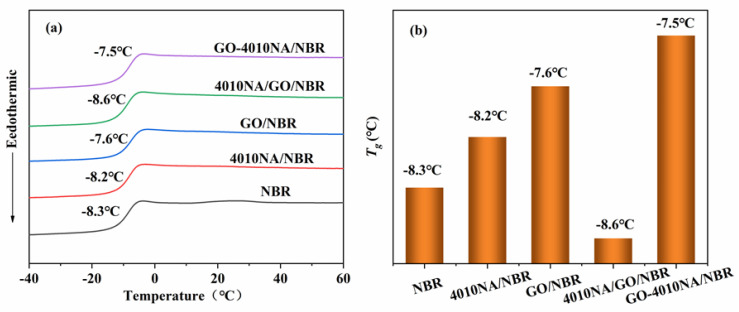
(**a**) DSC curves of different NBR composites and (**b**) the Tg of different NBR composites.

Figure 7 shows the DSC curves and the *T_g_* of different NBR composites. From Figure 7a, all the NBR composites have only one *T_g_*, indicating good compatibility between filler and NBR matrix [6]. The glass transition temperature (*T_g_*) of pure NBR was −8.3 °C, and the *T_g_* increases after the addition of 4010NA or GO as shown in Figure 7b, indicating that the interactions of the composites are enhanced. Compared with 4010NA/NBR composite, the *T_g_* of the GO-4010NA/NBR composite is increased to −7.5 °C. This increase is due to the H-bond network formed between GO-4010NA and NBR matrix [7,8]. The formation of H-bonds promotes the interaction of the composites and restricts the movement of NBR polymer chains, leading to an increase in the *T_g_*.

### 4.4. Dynamic Mechanical Properties of NBR Composites

**Figure 8:**
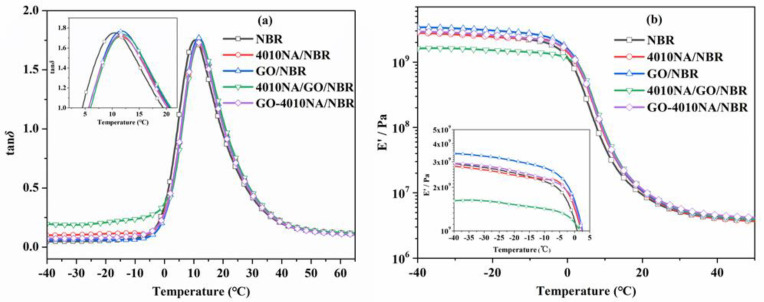
Temperature dependence of (**a**) the loss tangent (tan *δ*) values and (**b**) storage modulus for NBR composites.

Figure 8 shows the temperature dependence of the loss factor (tan *δ*) value and storage modulus (*E’*) of NBR composites. Table 2 shows the damping parameters of NBR composites. When the small molecule 4010NA is added to the NBR matrix, the tan *δ* peak value (denote as tan *δ_max_*) is 1.71 and the loss peak area TA is 29.58. When GO-4010NA was added, the tan *δ_max_* increased to 1.73 and TA increased to 30.05. The results show that the addition of GO-4010NA can improve the damping performance of NBR composites more than the addition of 4010NA, which is caused by the strong interactions between GO-4010NA and NBR. It indicates that more hydrogen bonds may be formed between GO-4010NA and NBR, thus improving the damping performance of the NBR composite.

### 4.5. Molecular Simulation Data Analysis

#### 4.5.1. Compatibility Analysis of 4010NA, GO, GO-4010NA and NBR

Improving the dispersity of filler is a key problem in the preparation of high-damping composites. The compatibility between filler and rubber can be evaluated by solubility parameters. The widely used solubility parameters are Hildebrand and Hansen solubility parameters [37].

(2) $$\delta_{T}=\sqrt{CED}=\sqrt{\frac{E_{c,T}}{V}}$$

where *δ_T_* is the Hildebrand solubility parameter, *V* is molar volume, and *E_C,T_* is cohesive energy. The non-bonding energy consists of three parts, namely dispersion, polarity, and hydrogen bonding. Hansen also proposed to divide the Hildebrand solubility parameter into three parts [38].

(3) $$\frac{E_{C,T}}{V}=\frac{E_{C,D}}{V}+\frac{E_{C,P}}{V}+\frac{E_{C,H}}{V}$$

where *E_C,D_*, *E_C,P_*, and *E_C,H_* represent dispersive, polar, and hydrogen bond cohesive energy, respectively. Combined with Equations (2) and (3), *δ_T_* can also be divided into three parts:

(4) $$\delta_{T}={\delta_{D}}^{2}+{\delta_{P}}^{2}+{\delta_{H}}^{2}$$

*δ_D_*, *δ_P_*, and *δ_H_* represent the dispersion, polar, and hydrogen bonding solubility parameters, known as Hansen solubility parameters. In the COMPASS force field, two energies are contained in *E_ele_*.

(5) $$E_{ele}=E_{C,P}+E_{C,H}$$

(6) $$E_{vdW}=E_{C,D}$$

Then, Equation (4) can be expressed as:

(7) $${\delta_{T}}^{2}={\delta_{vdW}}^{2}+{\delta_{ele}}^{2}$$

Therefore, in the COMPASS force field, the three-component Hansen solubility parameter is converted into a two-component solubility parameter (*δ_vdW_* and *δ_ele_*). It can be expressed by the following formula [39,40]:

(8) $$R=\sqrt{{\left(\delta_{vdW,A}-\delta_{vdW,B}\right)}^{2}+{\left(\delta_{ele,A}-\delta_{ele,B}\right)}^{2}}$$

**Figure 9:**
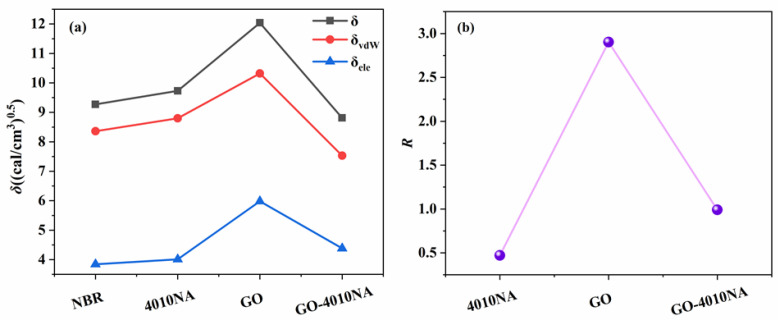
(**a**) Solubility parameter of NBR, 4010NA, GO, and GO-4010NA. (**b**) R value of NBR, 4010NA, GO, and GO-4010NA.

The compatibility of GO, 4010NA, and polymer is predicted by Cohesive Energy Density calculation *R* in the Forcite module. The physical significance of *R* is that the Hansen solubility of rubber is the distance from the spherical coordinate to the Hansen solubility parameter of filler. The smaller *R* is, the closer the solubility parameter is and the better the compatibility is [38,40]. The *δ*, *δ_vdW_*, and *δ_ele_* values of NBR, 4010NA, GO and GO-4010NA are calculated. Table 3 shows the *δ_vdW_* and *δ_ele_* values of NBR, 4010NA, GO, and GO-4010NA. Then, Equation (8) is used to calculate the value of *R*. The *δ* and *R* value of NBR, 4010NA, GO and GO-4010NA are shown in Figure 9. The results show that the *R* value increases first and then decreases, and the addition of GO made the *R* value increase, but the *R* value decreases after the addition of GO-4010NA, indicating that the GO graft 4010NA improves the compatibility with NBR, and there is good compatibility between NBR and GO-4010NA, which was consistent with the above DMA analysis.

#### 4.5.2. Charge Analysis of Atoms on Polar Functional Groups

**Figure 10:**
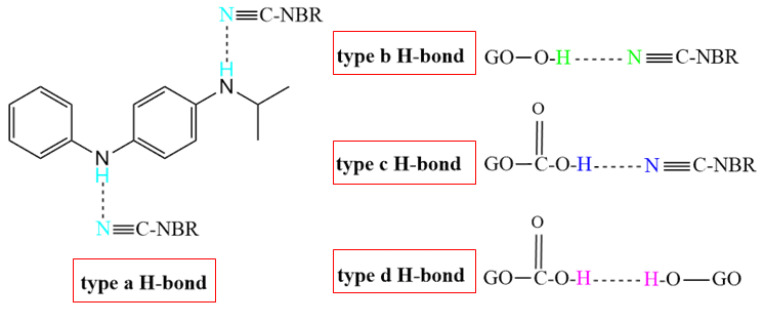
The four types of H-bonds might be formed in the NBR composites.

Table 4 shows the atomic charges on the polar functional groups of NBR, 4010NA, and GO molecules obtained by MD simulation. The stronger the electronegativity, the stronger the H-bond is expected to form. According to the atomic charge distribution, it can be predicted that four types of H-bonds may be formed in the NBR composites as shown in Figure 10. The type a H-bond may be between the -CN groups of NBR molecular chains and the -NH- groups of small molecules 4010NA, expressed as (4010NA)-NH…NC-(NBR). The type b H-bond may be between -CN groups of NBR molecular chains and -OH of GO, expressed as (GO)-OH…NC-(NBR). The type c H-bond may be between -CN groups of NBR molecular chains and -COOH groups of GO, expressed as (GO)-COOH…NC-(NBR). The type d H-bond may be between -OH groups of GO and -C-O-C- groups of GO, expressed as (GO)-O…HO-(GO). The specific types and numbers of H-bonds need to be further calculated.

#### 4.5.3. Accurate Statistics of Types and Number of H-Bonds

The types and numbers of H-bonds can be quantitatively calculated by MD simulation. As listed in Table 5, four types of H-bonds are formed in the NBR composites, including intermolecular H-bond interactions (such as H-bond types a, b, and c) and intramolecular H-bond interactions (such as H-bond type d). There is only one type of H-bond interaction in the 4010NA/NBR composite. With the addition of GO, it can be seen that the types and numbers of H-bonds increase significantly because there are more oxygen-containing functional groups on GO. The oxygen-containing functional groups on GO can not only form intermolecular H-bonds with NBR but also form intramolecular H-bonds between the oxygen-containing functional groups, so the addition of GO can significantly increase the types and numbers of H-bonds. Compared with the 4010NA/NBR composite, GO-4010NA/NBR composite has more H-bonds, indicating stronger interaction and better damping performance of the composite, which is consistent with the previous DMA experimental analysis.

#### 4.5.4. Binding Energy Analysis of NBR Composites

Binding energy (*E_binding_*) is defined as the negative value of intermolecular energy (*E_inter_*), which can reflect the mixing capacity and compatibility between each component. The larger *E_binding_* value is, the stronger the interface interaction is. If *E_binding_* is positive, the compatibility between two components is good. Formula (9) can be used to calculate [8]:

(9) $$E_{binding}=-E_{\mathrm{int}er}=-(E_{total}-E_{NBR}-E_{filler})$$

where *E_total_*, *E_NBR,_* and *E_filler_* are the total energies of the NBR composite, NBR, and the corresponding filler of composite, respectively.

**Figure 11:**
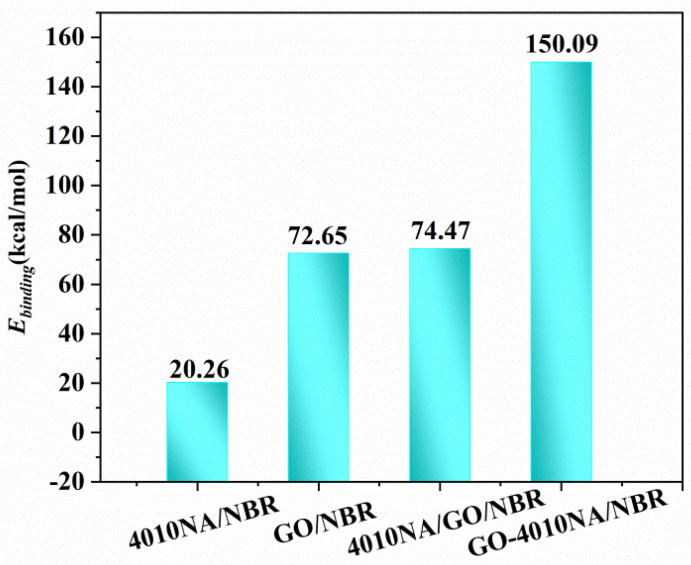
Binding energies of the different NBR composites.

The *E_binding_* of different NBR composites is shown in Figure 11. Compared with 4010NA/NBR, the addition of GO obviously increases the *E_binding_* of NBR composites, indicating a stronger interaction between GO and NBR and corresponding to the above H-bonds analysis. In particular, GO-4010NA/NBR composite has the largest binding energy, which is caused by the strong H-bonds of the composites, indicating the better damping performance of the composites.

#### 4.5.5. Free Volume Fraction (FFV) of Different NBR Composites

Free volume fraction (FFV) represents the stacking degree of polymer and is the percentage of free volume *V_f_* in the total volume *V*, which can be obtained from the expression below [39]:

(10) $$FFV=\frac{V-V*}{V}=\frac{V_{f}}{V}$$

where *V*, *V_f_*, and V* represent the total volume, free volume, and occupied volume of the composite, respectively.

**Figure 12:**
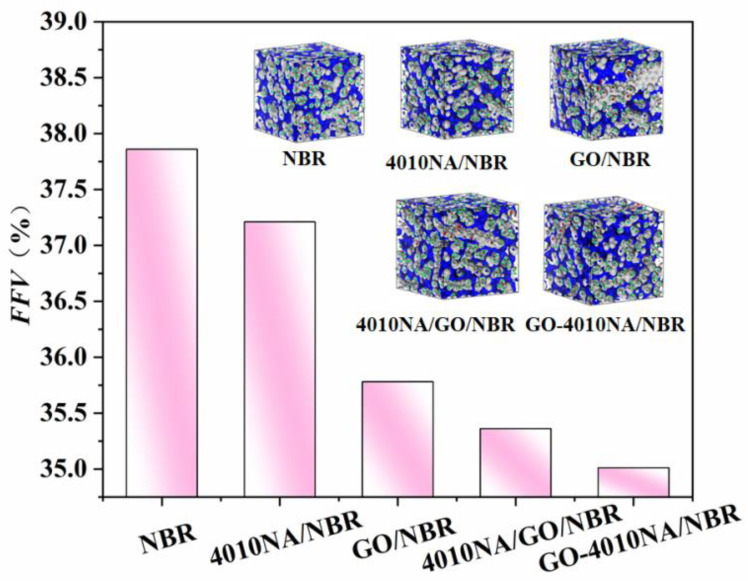
FFV of the different NBR composites by MD simulation, gray and blue areas represent the occupied and free volume, respectively.

The FFV of different NBR composites is shown in Figure 12. Compared with pure NBR, the FFV of 4010NA/NBR composite decreases. This is due to the formation of H-bonds, which restricts the free movement of NBR molecular chains. This is consistent with the previous analysis of H-bonds above. When GO is added into NBR, the FFV becomes smaller, mainly because a large number of hydrogen bonds are generated between GO and NBR, which makes the NBR molecular chain pile closer and the FFV decreases.

#### 4.5.6. Migration of Antioxidant 4010NA and Anti-Migration GO-4010NA

The mean square of the particle displacement in time t is called the mean square displacement (MSD). MSD can be used to describe the variation of molecular motion with time in the system [38]. Therefore, MSD can be used to study the migration characteristics of antioxidant molecules [39]. The equation for calculating MSD in MD simulation is [38]:

(11) $$MSD\left(t\right)=\frac{1}{N}{\sum_{i=1}^{N}\left[r_{i}\left(t\right)-r_{i}\left(0\right)\right]}^{2}$$

where, *N* is the total number of selected particles in the simulation system, and *r_i_*(*t*) and *r_i_*(*0*) are the final and initial position of atom *i* over the time interval *t*.

**Figure 13:**
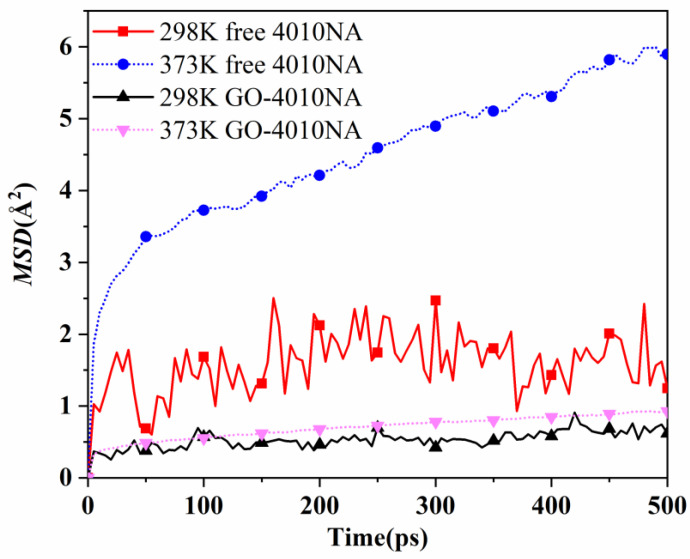
The MSD curves of 4010NA and GO-4010NA in NBR composites at different temperatures.

The movement of antioxidant 4010NA and GO-4010NA in 4010NA/NBR and GO-4010NA/NBR composites is simulated and analyzed at 298 K and 373 K, respectively. MSD curves of free and grafted 4010NA molecules are shown in Figure 13. The results show that MSD of both free 4010NA and grafted 4010NA increase with the increase of temperature, indicating that 4010NA was more mobile and migrated easily from the rubber matrix at a high temperature. At the same temperature, the MSD of free 4010NA is higher than that of GO-4010NA, indicating that the GO grafted 4010NA restricts the movement of 4010NA. The grafting method was effective to inhibit the migration of 4010NA.

## 5. Conclusions

In this work, a new type of antioxidant was successfully prepared by chemical grafting 4010NA to the GO surface by SOCl_2_. The effects of 4010NA and GO-4010NA on the compatibility and damping performance of NBR were studied by experimental and MD simulation methods. The main conclusions are as follows:

(1) DSC results show that NBR has good compatibility with 4010NA, GO, or GO-4010NA. Compared with 4010NA/NBR composite, the *T_g_* of the GO-4010NA/NBR composite is increased, which is due to more H-bond networks formed between GO-4010NA and the NBR matrix. DMA results show that the addition of GO-4010NA can increase the damping performance of NBR more effectively than the addition of 4010NA.
(2) Through MD simulation, the two-component solubility parameters between 4010NA, GO, GO-4010NA, and NBR matrix is calculated. Compared with 4010NA, the addition of GO-4010NA significantly improved the compatibility with NBR. The MD simulation is used to calculate the H-bonds, binding energy, and FFV of the NBR composites with different fillers. Compared with 4010NA/NBR composite, the GO-4010NA/NBR composite has more H-bonds, larger binding energy, and smaller FFV, indicating GO-4010NA/NBR composite has the better damping performance, which is consistent with the DMA results.
(3) Grafting 4010NA onto GO not only inhibits the migration of 4010NA but also improves the damping property of the NBR matrix. GO-4010NA is expected to be a functional filler for the preparation of high-damping NBR composites.

## Figures and Tables

**Table 1:** The experimental formulae of NBR composites ^a^.

Sample	Ingredients (phr)			
	NBR	GO	4010NA	GO-4010NA
NBR	100			
GO/NBR	100	1		
4010NA/NBR	100		0.1	
4010NA/GO/NBR	100	1	0.1	
GO-4010NA/NBR	100			1

^a^ Other rubber additives: ZnO, 5 phr; SA, 1 phr; D, 0.5 phr; DM, 0.5 phr; TMTD, 0.2 phr; S, 2 phr.

**Table 2:** Damping properties of the NBR composites.

Sample	Tan *δ* Peak Position(°C)	Tan *δ_max_*	Temperature Range > 0.3 (°C)			TA
			T_1_	T_2_	ΔT	
NBR	10.39	1.75	−0.56	34.04	34.60	31.12
4010NA/NBR	11.78	1.71	0.13	35.17	35.04	29.58
GO/NBR	11.73	1.77	0.55	34.98	34.43	31.30
4010NA/GO/NBR	11.95	1.74	−2.18	35.23	37.41	27.50
GO-4010NA/NBR	11.75	1.73	0.34	34.68	34.34	30.05

**Table 3:** Solubility parameters of NBR, 4010NA, GO, and GO-4010NA by MD simulation.

Materials	*δ_vdW_* (cal/cm^3^)^0.5^	Standard Error (cal/cm^3^)^0.5^	*δ_ele_* (cal/cm^3^)^0.5^	Standard Eror (cal/cm^3^)^0.5^
NBR	8.36	0.006	3.84	0.005
4010NA	8.80	0.026	4.01	0.029
GO	10.32	0.009	5.98	0.016
GO-4010NA	7.53	0.009	4.38	0.012

**Table 4:** Charges and forced field type of the atoms.

Polar Functional Groups	Atom	q(e)	Forced Field Type
-CN (NBR)	C	0.234	c2t
	N	−0.428	n1t
-NH (4010NA)	N	−0.373	n3h1
	H	0.353	h1n
-OH (GO)	H	0.410	h1o
	O	−0.570	o2h
-C-O-C (GO)	C	0.160	c44o
	O	−0.320	o2e
-CO_1_O_2_H (GO)	H	0.410	h1o
	O_2_	−0.455	o2c
	O_1_	−0.450	o1=
	C	0.495	c3′

**Table 5:** Numbers of H-bonds in NBR composites.

Sample	4010NA/NBR	GO/NBR	4010NA/GO/NBR	GO-4010NA/NBR
No. of type a H-bond	3	0	1	2
No. of type b H-bond	0	11	12	8
No. of type c H-bond	0	5	5	4
No. of type d H-bond	0	11	12	11
No. of total H-bonds	3	27	30	25

## Data Availability

The data presented in this study are available on request from the corresponding author.
